# Supplementary material for: Characterization of Volatile Profiles and Correlated Contributing Compounds in Pan-Fried Steaks from Different Chinese Yellow Cattle Breeds through GC-Q-Orbitrap, E-Nose, and Sensory Evaluation
Source: Molecules. 2022 Jun 2;27(11):3593. doi: 10.3390/molecules27113593 (PMC9182176; doi:10.3390/molecules27113593)
Supplement: Supplementary file 1 [file molecules-27-03593-s001.zip › molecules-1702765-supplementary.pdf]

molecules

MDPI

Article

Characterization of Volatile Profiles and Correlated Contributing Compounds in Pan-Fried Steaks from Different Chinese Yellow Cattle Breeds through GC-Q-Orbitrap, E-Nose, and Sensory Evaluation

Meng Wei [1,2], Xiaochang Liu [1], Peng Xie [1], Yuanhua Lei [1], Haojie Yu [1], Aiyun Han [2], Libin Xie [2], Hongliang Jia [3], Shaohua Lin [3], Yueyu Bai [4,5], Baozhong Sun [1] and Songshan Zhang [1,*]

1 Institute of Animal Sciences, Chinese Academy of Agricultural Sciences, Beijing 100193, China; weimeng@caas.cn (M.W.); lxc_cau@163.com (X.-c.L.); seulbird@163.com (P.X.); leiyuanhua-1@163.com (Y.-h.L.); haojie-yu@foxmail.com (H.-j.Y.); baozhongsun@163.com (B.-z.S.)
2 Chemical Engineering Institute, Shijiazhuang University, Shijiazhuang 050035, China; irene0001@126.com (A.-y.H.); xielibin827@163.com (L.-b.X.)
3 Department of Food and Biological Engineering, Beijing Vocational College of Agriculture, Beijing 102442, China; jiahongliang@bvca.edu.cn (H.-l.J.); lsh_hp@sina.com (S.-h.L.)
4 Henan Animal Health Supervision, Zhengzhou 450000, China; baiyueyu666@sina.com
5 School of Agricultural Sciences, Zhengzhou University, Zhengzhou 450000, China
* Correspondence: zhangsongshan@caas.cn

Table S1. Qualitative and quantitative analyses of volatile organic compounds (VOCs) of pan-fried steaks from different yellow cattle breeds.

Compounds Information					Relative contents of VOCs (μg/kg)						
Types of VOCs	NO.	Component Name	Calculated RI[x]	Library RI[y]	BH	JX	YL	WS	XJ	PL	SEM
Aldehydes	1	Acetaldehyde	706	702	462.92b	780.86a	551.79b	771.33a	753.15a	755.44a	51.67
	2	Butanal	906	877	74.08c	115.36b	86.81c	126.48ab	130.97ab	145.60a	9.06
	3	3-Methyl-butanal	921	923	53.75c	90.01c	51.31c	73.52c	139.77b	206.48a	12.77
	4	Pentanal	983	991	78.78b	86.59b	200.12ab	376.03a	275.22a	327.56a	56.84
	5	Hexanal	1086	1098	2591.35b	2864.51b	7626.23ab	12495.07a	9546.86ab	11539.15a	2224.46
	6	Heptanal	1190	1192	619.65c	475.61c	1257.02bc	3104.26a	1446.47bc	2147.91ab	411.08
	7	Octanal	1295	1298	492.43c	465.04c	1266.89b	2348.26a	904.80bc	1528.70b	215.90
	8	(E)-2-Heptenal	1332	1335	22.33b	26.72b	57.74ab	93.63a	86.05a	81.74a	14.16
	9	Nonanal	1400	1402	1984.34d	1672.72d	6919.63b	12250.18a	3607.64cd	6133.55bc	1018.44
	10	(E)-2-Octenal	1438	1441	161.06b	193.96b	450.27ab	641.19a	565.28a	660.01a	102.25
	11	Decanal	1506	1508	84.27c	85.31c	186.05b	311.87a	159.36bc	158.39bc	28.54
	12	Benzaldehyde	1537	1541	14837.13d	21394.33bc	18745.57cd	27236.65ab	24484.88bc	32321.44a	1989.02
	13	(E)-2-Nonenal	1545	1547	223.84b	218.94b	419.57b	838.83a	555.05ab	932.20a	127.47
	14	(E)-2-Decenal	1654	1644	34.38d	34.46d	93.54bc	164.27a	61.97cd	113.04b	13.68
	15	Benzeneacetaldehyde	1657	1661	2451.95c	3139.09bc	2812.43bc	4040.99b	5624.90a	6305.57a	422.71
	16	4-Ethyl-benzaldehyde	1724	1721	20.00d	31.59cd	63.79bcd	95.38b	81.66bc	150.61a	17.13
	17	E-Citral	1742	1744	1.06d	1.17d	13.26b	32.94a	2.60c	3.83c	0.46
	18	(E)-2-Undecenal	1762	1751	125.58d	126.48d	285.71bc	411.62a	212.66cd	374.47ab	36.59
	19	(E,Z)-2,4-Decadienal	1775	1754	11.68b	13.37b	27.58ab	45.81a	34.29a	44.36a	5.64
	20	(E,E)-2,4-Decadienal	1822	1823	15.60b	17.36b	29.09ab	41.29a	47.60a	48.90a	6.76
	21	4-Pentyl-benzaldehyde	2023	2003	12.41d	22.57cd	40.01bc	50.35b	57.63b	84.44a	7.89
	22	4-Methoxy-benzaldehyde	2045	2011	19.18d	25.02cd	73.96b	166.36a	37.90c	30.57cd	4.67
Alcohols	23	Isopropyl alcohol	931	927	201.51b	174.37b	2792.24a	216.41b	286.30b	191.46b	35.35
	24	1-Hexanol	1352	1355	126.10	103.59	105.78	182.05	83.55	137.40	28.44

	25	1-Octen-3-ol	1449	1451	546.40c	796.94c	2023.49ab	3222.68a	1682.69bc	2378.40ab	375.12
	26	1-Octanol	1558	1557	321.35cd	269.12d	1078.54b	1619.16a	466.41cd	746.45bc	135.18
	27	(Z)-5-Octen-1-ol	1617	1619	94.50c	123.40c	301.47b	498.21a	229.62bc	340.10ab	52.19
	28	alpha-Terpineol	1703	1706	7.18d	5.96d	16.37b	41.36a	10.79c	7.10d	0.87
	29	Benzenemethanol	1885	1890	53.18b	60.34b	81.88b	1016.79a	67.36b	65.14b	37.73
	30	2-Ethyl-hexanoic acid	1953	1964	37.19	70.81	125.19	54.25	4.86	86.57	27.85
Fatty acid	31	Nonanoic acid	2171	2171	7812.41a	9573.99a	8646.77a	10375.99a	94.26b	11852.53a	1695.85
	32	Decanoic acid	2277	2289	132.77a	149.64a	136.16a	174.71a	21.03b	184.82a	18.77
	33	Methyl-pyrazine	1271	1266	63.40c	164.25b	83.02c	89.44c	217.24a	223.55a	10.69
	34	2,5-Dimethyl-pyrazine	1327	1320	908.38c	2500.21b	1251.54c	1522.94c	3034.71b	3791.42a	198.72
	35	2,6-Dimethyl-pyrazine	1333	1328	318.82c	948.45b	417.08c	483.07c	1244.16a	1259.11a	70.00
	36	2,3-Dimethyl-pyrazine	1352	1344	26.34d	66.19b	39.29cd	44.37c	87.65a	95.30a	4.70
	37	2-Ethyl-6-methyl-pyrazine	1390	1387	51.92c	162.97b	90.28c	90.58c	247.85a	271.42a	17.14
Nitrogen-containing heterocyclic compounds	38	2-Ethyl-5-methyl-pyrazine	1397	1387	8.80d	26.27c	12.39d	16.22d	36.11b	47.90a	2.56
	39	Trimethyl-pyrazine	1408	1402	698.94c	2252.85b	1124.88c	1333.00c	2753.97b	3548.70a	222.68
	40	2,3-Dimethyl-3-ethylpyrazine	1450	1469	639.77c	1551.66b	1145.15bc	1571.32b	1734.43b	3044.94a	193.99
	41	2,3-Dimethyl-5-ethylpyrazine	1467	1469	139.38d	434.30bc	230.97d	290.20cd	548.71b	847.38a	59.01
	42	2,5-Dimethyl-3-(3-methylbutyl)-pyrazine	1663	1666	16.82b	23.55b	18.84b	33.70b	33.12b	75.53a	6.19
	43	6,7-Dihydro-2,5-dimethyl-5H-cyclopentapyrazine	1685	1672	1.24c	3.89b	1.52c	2.57c	4.42b	5.79a	0.42
	44	2-Acetylpyrrole	1981	1973	291.74c	892.01ab	283.50c	376.75c	1081.83a	750.19b	78.68
	45	Methanethiol	677	692	330.79c	660.03b	310.79c	1153.21a	668.15b	1089.64a	103.67
	46	Carbon disulfide	734	735	259.90b	316.99b	157.40b	158.36b	229.24b	2008.83a	146.49
	47	Dimethyltrisulfide	1395	1398	7.96ab	8.62a	4.37c	5.74bc	6.42abc	8.39ab	0.85
Sulfur-containing compounds	48	Methional	1465	1469	53.57cd	35.48d	35.41d	62.04c	95.28b	121.44a	7.36
	49	2-Acetylthiazole	1660	1664	28.23c	47.72b	32.17bc	37.84bc	76.66a	75.04a	4.97
	50	2-Acetyl-2-thiazoline	1773	1778	140.19d	669.05a	299.62c	225.62cd	611.36a	407.81b	33.87
	51	Dimethyl sulfone	1913	1920	17.57c	366.34b	34.22c	62.76c	81.52c	687.29a	72.00
	52	Benzothiazole	1977	1981	31.18c	33.85c	40.31bc	62.53a	48.79b	32.46c	3.96
Oxygen-	53	2-Pentyl-furan	1237	1241	121.18b	183.14b	347.92ab	659.81a	436.56ab	585.80a	97.13

containing heterocyclic compounds	54	Furfural	1473	1477	309.97bc	318.73bc	252.72c	257.16c	375.59ab	457.66a	27.64
	55	Butyrolactone	1645	1652	10.89d	22.98ab	15.40cd	19.00bc	27.68a	21.89ab	1.94
	56	2-Furanmethanol	1663	1668	14.95a	14.77a	5.46c	8.17bc	11.75ab	10.22b	1.25
	57	gamma-Octalactone	1934	1938	8.89a	8.94a	4.37b	5.98b	5.83b	6.83ab	27.55
	58	Ethyl maltol	2025	2050	6.00bcd	4.45cd	3.66d	10.18a	8.19ab	6.42bc	0.74
Esters	59	Octanoic acid, methyl ester	1395	1396	130.35b	107.18b	15.55c	43.01c	0.33c	256.22a	14.92
	60	alpha-Terpinenyl Acetate	1708	1692	6.80c	5.06c	11.12b	51.55a	7.42c	8.07bc	1.13
Ketones	61	2-Butanone	905	908	89.64c	135.71b	100.78c	146.57ab	153.80ab	175.02a	10.63
	62	2-Pentanone	980	981	10.16	702.98	248.64	22.77	39.03	544.23	272.51
	63	2-Heptanone	1186	1182	41.18b	45.96b	78.24ab	120.94a	91.65ab	114.49a	16.46
	64	Acetoin	1291	1298	1972.04c	3855.65b	3409.76b	3471.91b	5672.99a	6381.39a	299.41
	65	2,5-Octanedione	1328	1319	357.12c	483.96bc	1063.04abc	2632.37a	2227.80ab	1959.56abc	528.03
	66	6-Methyl-5-hepten-2-one	1343	1346	13.71d	17.53d	38.59bc	81.73a	28.71c	44.81b	3.37
Hydrocarbons	67	Methyl-cyclopentane	675	975	1507.13d	2372.67bc	2042.57bc	2462.13b	1989.99c	3005.83a	130.95
	68	Benzene	946	957	26.53d	63.10d	450.39b	227.50c	8.30d	712.05a	31.34
	69	Toluene	1045	1049	2749.03c	3002.99bc	1815.72c	2395.32c	4207.19b	8656.97a	406.51
	70	Undecane	1101	1100	63.88b	58.68b	38.88bc	53.36b	1.30c	178.54a	14.52
	71	Ethylbenzene	1133	1129	1006.73c	854.48cd	405.19d	586.57cd	1528.47b	2903.28a	157.66
	72	p-Xylene	1142	1138	1819.63bc	1506.62cd	693.13d	1091.86cd	2612.19b	5261.35a	286.26
	73	m-Xylene	1148	1143	9309.34c	8216.51c	3750.99d	5546.81cd	14191.27b	26161.39a	1370.99
	74	1-Butyl-cyclohexene	1165	1187	28.18b	11.71bc	13.61bc	19.47bc	5.45d	55.45a	6.14
	75	o-Xylene	1193	1199	1795.84c	1564.96cd	729.02d	1091.30cd	2659.37b	4920.96a	265.31
	76	dl-Limonene	1206	1211	95.25c	71.59c	61.10c	179.89b	273.73a	272.94a	19.26
	77	Styrene	1266	1270	270.99c	255.98c	191.05c	252.20c	722.88b	1434.76a	97.93
	78	Cymene	1279	1269	21.00c	19.19c	13.66c	25.98c	43.80b	77.96a	4.74
	79	Tridecane	1303	1300	201.15b	153.69b	77.35c	211.00b	17.78d	282.10a	19.18
	80	3-Pentyl-cyclohexene	1310	1289	43.56bc	40.63bc	26.58c	54.78b	0.00d	108.12a	6.97
	81	Tetradecane	1403	1399	293.40b	203.81bc	212.67bc	402.85a	143.89c	389.49a	29.33
	82	1,3-bis(1,1-Dimethylethyl)-benzene	1435	1427	1.18c	1.02c	1.41bc	28.55a	2.26bc	6.91b	1.71

	83	1,2,3,5-Tetramethyl-benzene	1449	1422	19.66c	16.76c	71.77a	51.26b	41.67b	45.63b	3.24
	84	trans-Caryophyllene	1616	1595	5.95c	4.67c	4.48c	39.74a	29.45b	24.32b	2.02
	85	Heptadecane	1704	1699	10.58d	11.77cd	15.91bc	21.37a	10.72d	16.88b	1.40
	86	Naphthalene	1762	1764	460.64b	207.59c	251.41c	621.15a	457.38b	386.35b	32.75
	87	Octadecane	1793	1799	25.08c	9.45d	36.86b	53.58a	13.05d	31.53bc	2.81
	88	Trichloromethane	1024	1022	342.94bc	365.70bc	234.49c	313.91c	575.03b	1722.66a	78.31
	89	Chloro-benzene	1223	1233	1.65c	0.40c	0.32c	1.19c	34.76a	3.98b	0.45
Others	90	N,N-dibutyl-formamide	1779	1757	328.56b	275.72b	549.36a	543.77a	569.12a	652.44a	53.48
	91	Anethole	1841	1817	4.71d	4.42d	8.47bc	24.46a	6.28cd	9.07b	0.75
	92	3-Methyl-phenol	2091	2099	58.68d	58.38d	107.68a	97.27ab	76.14bc	77.41bc	6.94

[x] Retention index (RI) calculated according to the retention times of n-alkanes standard (C_6-C_{30}).
[y] Retention index (RI) matched from NIST and WILEY libraries.
a–d different letters in the same row indicate significant differences at $p < 0.05$.
SEM, standard error of the mean.
BH, Bohai Black Cattle; JX, Jiaxian Red Cattle; YL, Yiling Cattle; WS, Wenshan Cattle; XJ, Xinjiang Brown Cattle; PL, Pingliang Red Cattle.

Table S2. Volatiles of odor activity values (OAVs) ≥ 1 of pan-fried steaks from different yellow cattle breeds.

Compounds Information			OAV					
**NO.**	**Component Name**	**Threshold(μg/kg)**	**BH**	**JX**	**YL**	**WS**	**XJ**	**PL**
1	Butanal	15.90	4.66	7.26	5.46	7.95	8.24	9.16
2	3-Methyl-butanal	13.00	4.13	6.92	3.95	5.66	10.75	15.88
3	Pentanal	100.00	0.79	0.87	2.00	3.76	2.75	3.28
4	Hexanal	750.00	3.46	3.82	10.17	16.66	12.73	15.39
5	Heptanal	550.00	1.13	0.86	2.29	5.64	2.63	3.91
6	Octanal	248.00	1.99	1.88	5.11	9.47	3.65	6.16
7	(E)-2-Heptenal	40.00	0.56	0.67	1.44	2.34	2.15	2.04
8	Nonanal	260.00	7.63	6.43	26.61	47.12	13.88	23.59
9	(E)-2-Octenal	0.20	805.31	969.79	2251.37	3205.95	2826.38	3300.03
10	Benzaldehyde	4600.00	3.23	4.65	4.08	5.92	5.32	7.03
11	(E)-2-Nonenal	0.69	324.41	317.30	608.08	1215.69	804.42	1351.02
12	Benzeneacetaldehyde	30.00	81.73	104.64	93.75	134.70	187.50	210.19
13	(E)-2-Undecenal	1.40	89.70	90.35	204.08	294.01	151.90	267.48
14	(E,Z)-2,4-Decadienal	0.07	166.90	190.99	393.99	654.37	489.81	633.65
15	(E,E)-2,4-Decadienal	10.00	1.56	1.74	2.91	4.13	4.76	4.89
16	4-Methoxy-benzaldehyde	27.00	0.71	0.93	2.74	6.16	1.40	1.13
17	1-Octen-3-ol	100.00	5.46	7.97	20.23	32.23	16.83	23.78
18	(Z)-5-Octen-1-ol	6.00	15.75	20.57	50.25	83.03	38.27	56.68
19	2-Ethyl-6-methyl-pyrazine	40.00	1.30	4.07	2.26	2.26	6.20	6.79
20	2,3-Dimethyl-3-ethylpyrazine	1.00	639.77	1551.66	1145.15	1571.32	1734.43	3044.94
21	2,3-Dimethyl-5-ethylpyrazine	1.00	139.38	434.30	230.97	290.20	548.71	847.38
22	2-Acetylpyrrole	100.00	2.92	8.92	2.84	3.77	10.82	7.50
23	Methanethiol	4.00	82.70	165.01	77.70	288.30	167.04	272.41

24	Carbon disulfide	5.00	51.98	63.40	31.48	31.67	45.85	401.77
25	Dimethyltrisulfide	0.10	79.62	86.21	43.71	57.41	64.17	83.87
26	3-(Methylthio)-propanal	1.80	29.76	19.71	19.67	34.47	52.93	67.47
27	2-Acetylthiazole	10.00	2.82	4.77	3.22	3.78	7.67	7.50
28	2-Acetyl-2-thiazoline	1.30	107.84	514.66	230.48	173.55	470.28	313.70
29	Dimethyl sulfone	175.00	0.10	2.09	0.20	0.36	0.47	3.93
30	2-Pentyl-furan	14.50	8.36	12.63	23.99	45.50	30.11	40.40
31	p-Xylene	1000.00	1.82	1.51	0.69	1.09	2.61	5.26
32	m-Xylene	1100.00	8.46	7.47	3.41	5.04	12.90	23.78
33	o-Xylene	1800.00	1.00	0.87	0.41	0.61	1.48	2.73
34	Trichloromethane	307.00	1.12	1.19	0.76	1.02	1.87	5.61
